# Self-Reiki, Consideration of a Potential Option for Managing Chronic Pain during Pandemic COVID-19 Period

**DOI:** 10.3390/medicina57090867

**Published:** 2021-08-25

**Authors:** Maxime Billot, Maeva Daycard, Philippe Rigoard

**Affiliations:** 1 PRISMATICS Lab (Predictive Research in Spine/Neuromodulation Management and Thoracic Innovation/Cardiac Surgery), Poitiers University Hospital, 86021 Poitiers, France; philippe.rigoard@chu-poitiers.fr; 2 Eveil: L’équilibre par les Mains, 87000 Poitiers, France; maevaday@gmail.com; 3 Department of Spine Surgery and Neuromodulation, Poitiers University Hospital, 86021 Poitiers, France; 4 Institut Pprime UPR 3346, CNRS—Université de Poitiers—ISAE-ENSMA, 86360 Chasseneuil-du-Poitou, France

**Keywords:** complementary and alternative medicine, therapeutic touch, healing touch, biofield therapy, pain treatment, energy, self-treatment, lockdown

## Abstract

While the world faces an unprecedented situation with the pandemic, other chronic diseases such as chronic pain continue to run their course. The social distancing and restrictive displacement imposed by the pandemic situation represents a new barrier to access to pain management and tends to reinforce chronification process. Given this context, complementary and alternative medicine (CAM) might offer new opportunities to manage CP, notably with a hand-touch method, such as self-Reiki therapy. Although Reiki administered by a practitioner has shown promising results to reduce pain and psychological distress, and to improve quality of life, self-Reiki practice needs evidence-based medicine to be disseminated. Overall, self-Reiki could bring positive results in addition to, and without interfering with, conventional medicine approaches in patients experienced chronic pain.

The fast spread of coronavirus 2019 (COVID-19) throughout the world has compelled the World Health Organization (WHO) to declare the outbreak as an international public health emergency (30 January 2020) and then a pandemic (11 March 2020) [1]. To contain the COVID-19 pandemic, recommendations have ranged from social distancing and restrictive displacement to containment. At the same time, just like all other chronic diseases, chronic pain (CP) continued to run its course. Defined by the International Association of the Study of Pain (IASP) as a pain that persists or recurs longer than 3 months [2,3], CP nowadays affects more than 2 billion people, and represents both societal and financial burdens amounting to several billion dollars/euros [4,5]. CP impacts physical and mental health as well as social factors [6], and consequently alters quality of life [7,8,9,10,11,12]. The social distancing imposed by the pandemic situation represents a new barrier, aggravating levels of social isolation in the CP population [13]. While the first indication to manage pain lies in consultation of a medical and/or paramedical professional, restrictive displacement and containment not only do not facilitate access to healthcare, but also tends to reinforce the chronification process. The COVID-19 pandemic has compelled the medical and paramedical community to redefine their practices, notably through technology such as teleconsultation. While teleconsultation can help to identify illness, it cannot be fully effective when face-to-face health care is required. In this challenging situation, complementary and alternative medicine (CAM) are booming and might offer new opportunities to manage CP, notably with a hand-touch method such as Reiki therapy.

Reiki was born in early 20th century Japan following the inspiring practice of Mikao Usui, a lifelong practitioner of Tendai who provided health care by spreading energy with his hands to a recipient [14]. It was only after Takata’s attempt to disseminate the practice of Reiki at the end of the 1970’s that Reiki was democratized. Reiki is nowadays officially recognized and recommended by the National Center for Complementary and Alternative Medicine in USA [15], and by the National Health Service and The Prince of Wales’s Foundation for Integrated Health in the UK [16]. Traditionally, Reiki treatment is a hands-on treatment provided by light touch, lasting from 45 to 75 min, administered to a fully clothed and quiet recipient who is comfortably seated or lying down [14]. The principle of Reiki treatment is that the practitioner, acting as a channel for universal energy, transfers energy to a recipient. Typically, a Reiki session consists of at least 12 standardized positions on the head and in the seven main energy centers (called chakras), and could also be directly targeted to the painful or disabled area. Reiki has been recognized as a biofield therapy category (a concept defined as “a massless field, not necessarily electromagnetic, that surrounds and permeates living bodies and affects the body” [17]) that could benefit global health and/or specific symptoms such as pain. Reiki practice considers three degrees (first, second, and Master), each one of which provides a scope of practice with major interest in self-treatment and light touch treatment for others. While usual Reiki therapy involves a practitioner guiding energy to a living receiver (hetero-Reiki), it is also possible to be the practitioner and the recipient at the same time (self-Reiki). To date, scientific evidence has mainly focused on hetero-Reiki [18].

Investigating the effect of Reiki on autonomic dysfunction through heart rate variability (HRV), Friedman et al. [19] reported that Reiki significantly improved HRV, which is known to be protective, in recovery from acute coronary syndrome. In addition, the authors showed that Reiki treatment was associated with decreased negative emotional states (stressed, angry, sad, frustrated, worried, scared, and anxious) and increased positive emotional states (happy, relaxed, and calm). In the most recent meta-analysis, Dogan [20] reported, though four randomized controlled trials (RCTs) and 212 patients, a large effect size of Reiki therapy on pain relief. However, this result should be carefully interpreted since they also reported significant heterogeneity. This limitation was reinforced by the small number of RCTs, methodological concerns, and the lack of standardized Reiki therapy program (i.e., number of sessions, frequency, duration, etc.). Overall, the seven reviews [20,21,22,23,24,25,26] available in the literature failed to establish strong evidence of the effectiveness of Reiki therapy to relieve pain in various CP conditions. Although a high level of evidence through RCTs is still needed to establish Reiki therapy as an effective practice, people having experienced hetero- or self-Reiki have reported globally positive pain management. In addition to pain treatment, it has been reported that Reiki could be helpful to manage psychological components of CP disease.

The literature has indicated positive effects of Reiki therapy, including decreased anxiety in healthy persons [27], people with various CP conditions [28], people with abdominal hysterectomies [29], women with breast biopsy [30], people with stage I to IV cancer [31] and community-dwelling older adults [32]. Similarly, the positive effects of Reiki therapy on depression in groups with various CP conditions [28], depressive conditions [33], women with breast biopsies [30], and elderly people living in community housing or nursing homes have been reported [32,34], while two studies indicated no effects in post-stroke patients [35] and patients with prostate cancer treated by radiation [36]. Particularly noteworthy is the reported effectiveness of Reiki therapy in 29 patients identified as depressive (19–78 years) who received Reiki therapy session in comparison with those having received placebo treatment [33]. In addition, two recent reviews supported the argument that Reiki therapy is valuable as a complementary therapy to manage anxiety and depression symptoms [22,25], whereas another review emphasized information insufficient to delineate the exact effect of Reiki therapy on psychological distress [37]. While patients mainly used the words ‘anxious’, ‘fearful’, ‘irritable’, ‘restless’, ‘stressed’, or ‘pain’ before the Reiki treatment, they mostly used the word ‘calm’ after the intervention [38]. By the same token, while little clinical-based evidence is available [14], all Reiki practitioners have experienced well-being by self-Reki practice. Given that self-Reiki can be a valuable tool and a special personal gift for management of CP in this time of pandemic, how can this practice be demystified and promoted?

It is worthwhile to note that Reiki is accessible for everyone without any preliminary restrictions. The technique can be taught by a Master of Reiki who provides the tools necessary to use one’s hands and spread energy on the pain location by oneself. Self-treatment is fundamental for all practitioners from the beginner to the Master of Reiki. Self-treatment is easy to learn and easy to practice since it involves softly placing hands on different parts of the body [39], especially the painful ones. Besides, the ability to discern the energy by the person receiving energy or, in this case, the practitioner, does not seem to be related to the effectiveness of the treatment. The sensations felt by the practitioner (hot, cold, or tingling sensation) could guide and help them to hold the hands in a location or to move them when they have faded. When no sensation is felt, it is recommended to wait at least 3 min by hand position before to removing them. Furthermore, self-Reiki can be practiced at any time of the day from waking up to right before sleeping.

The democratization of this practice could be achieved through wide dissemination. The most effective way to understand Reiki is to receive a treatment or to practice it. To achieve this goal, trained nurses and medical doctors can be valuable vehicles to promoters of Reiki in addition to the specialized Reiki therapists. Reiki is already present in several hospitals worldwide, a factor that may also help to demystify the practice [40]. The next step should involve setting up learning sessions with patients themselves, for instance by integrating Reiki in the medical pathway of the therapeutic education activities (or through webinar). Inspired by other alternative approaches, such as self-hypnosis performed at home [41,42], self-Reiki could be administered as a preventive/curative way in patient suffering from chronic pain. In addition, in a pilot study, Chen et al. [43] suggested that Reiki delivered by family caregivers to cancer patients in a home setting was beneficial in reducing cancer symptoms and enhancing health-related outcomes. Obviously, research must carry on with its attempts to demonstrate the effectiveness and limitations of this practice through randomized controlled trials with a high level of evidence, with special attention being paid to potential responder characteristics.

In conclusion, the pandemic situation represents a new and challenging condition for patients presenting with CP. Complementary and alternative medicine, especially Reiki practice, has shown promising results to reduce pain and psychological distress, and to improve quality of life. Although self-Reiki practice has not been yet supported by strong evidence-based medicine, it is safe to assume that it could bring positive results and could be considered as an option for managing chronic pain in addition to, and without interfering with, conventional medicine approaches, notably during the pandemic period.

## Data Availability

Not applicable.

## References

[1] World Health Organization (2020). Mental Health and Psychosocial Considerations during the COVID-19 Outbreak, 18 March 2020.

[2] Merskey H., Bogduk N. (1994). Task Force on Taxonomy. Classification of Chronic Pain: Descriptions of Chronic Pain Syndromes and Definitions of Pain Terms.

[3] Treede R.-D., Rief W., Barke A., Aziz Q., Bennett M.I., Benoliel R., Cohen M., Evers S., Finnerup N.B., First M.B. (2019). Chronic pain as a symptom or a disease: The IASP Classification of Chronic Pain for the International Classification of Diseases (ICD-11). Pain.

[4] Mills S.E., Nicolson K.P., Smith B. (2019). Chronic pain: A review of its epidemiology and associated factors in population-based studies. Br. J. Anaesth..

[5] Gaskin D.J., Richard P. (2012). The Economic Costs of Pain in the United States. J. Pain.

[6] Naiditch N., Billot M., Moens M., Goudman L., Cornet P., Le Breton D., Roulaud M., Ounajim A., Page P., Lorgeoux B. (2021). Persistent Spinal Pain Syndrome Type 2 (PSPS-T2), a Social Pain? Advocacy for a Social Gradient of Health Approach to Chronic Pain. J. Clin. Med..

[7] Blyth F.M., Noguchi N. (2017). Chronic musculoskeletal pain and its impact on older people. Best Pr. Res. Clin. Rheumatol..

[8] Boutron I., Rannou F., Jardinaud-Lopez M., Meric G., Revel M., Poiraudeau S. (2008). Disability and quality of life of patients with knee or hip osteoarthritis in the primary care setting and factors associated with general practitioners’ indication for prosthetic replacement within 1 year. Osteoarthr. Cartil..

[9] Schmader K.E. (2002). Epidemiology and Impact on Quality of Life of Postherpetic Neuralgia and Painful Diabetic Neuropathy. Clin. J. Pain.

[10] Colloca L., Ludman T., Bouhassira D., Baron R., Dickenson A.H., Yarnitsky D., Freeman R., Truini A., Attal N., Finnerup N. (2017). Neuropathic pain. Nat. Rev. Dis. Prim..

[11] Wittkopf P.G., Zomkowski K., Cardoso F.L., Sperandio F.F. (2017). The effect of chronic musculoskeletal pain on several quality of life dimensions: A critical review. Int. J. Ther. Rehabil..

[12] Jensen M.P., Chodroff M.J., Dworkin R.H. (2007). The impact of neuropathic pain on health-related quality of life: Review and implications. Neurology.

[13] Hruschak V., Flowers K.M., Azizoddin D.R., Jamison R.N., Edwards R.R., Schreiber K.L. (2021). Cross-sectional study of psychosocial and pain-related variables among patients with chronic pain during a time of social distancing imposed by the coronavirus disease 2019 pandemic. Pain.

[14] Miles P., True G. (2003). Reiki—Review of a biofield therapy history, theory, practice, and research. Altern. Ther. Health Med..

[15] National Center for Complementary and Integrative Health Statistics on Complementary and Integrative Health Approaches. NCCIH n.d. https://www.nccih.nih.gov/research/statistics-on-complementary-and-integrative-health-approaches.

[16] Tavares M. (2003). National Guidelines for the Use of Complementary Therapies in Supportive and Palliative Care.

[17] Rubik B., Muehsam D., Hammerschlag R., Jain S. (2015). Biofield Science and Healing: History, Terminology, and Concepts. Glob. Adv. Health Med..

[18] Herron-Marx S., Price-Knol F., Burden B., Hicks C. (2008). A Systematic Review of the Use of Reiki in Health Care. Altern. Complement. Ther..

[19] Friedman R.S., Burg M.M., Miles P., Lee F., Lampert R. (2010). Effects of Reiki on Autonomic Activity Early After Acute Coronary Syndrome. J. Am. Coll. Cardiol..

[20] Doğan M.D. (2018). The effect of reiki on pain: A meta-analysis. Complement. Ther. Clin. Pr..

[21] Lee M.S., Pittler M.H., Ernst E. (2008). Effects of reiki in clinical practice: A systematic review of randomised clinical trials. Int. J. Clin. Pr..

[22] Thrane S., Cohen S.M. (2014). Effect of Reiki Therapy on Pain and Anxiety in Adults: An In-Depth Literature Review of Randomized Trials with Effect Size Calculations. Pain Manag. Nurs..

[23] Vandervaart S., Gijsen V.M.G.J., de Wildt S., Koren G. (2009). A Systematic Review of the Therapeutic Effects of Reiki. J. Altern. Complement. Med..

[24] Vitale A. (2007). An Integrative Review of Reiki Touch Therapy Research. Holist. Nurs. Pr..

[25] McManus D.E. (2017). Reiki Is Better Than Placebo and Has Broad Potential as a Complementary Health Therapy. J. Evid. Based Integr. Med..

[26] Billot M., Daycard M., Wood C., Tchalla A. (2019). Reiki therapy for pain, anxiety and quality of life. BMJ Support. Palliat. Care.

[27] Rnc D.W.W., Engebretson J. (2001). Biological correlates of Reiki Touchsm healing. J. Adv. Nurs..

[28] Dressen L.J., Singg S. (1998). Effects of reiki on pain and selected affective and personality variables of chronically ill patients. Subtle Energ. Energy Med..

[29] Vitale A.T., O’Connor P.C. (2006). The effect of Reiki on pain and anxiety in women with abdominal hysterectomies: A quasi-experimental pilot study. Holist. Nurs. Pr..

[30] Potter P.J. (2007). Breast Biopsy and Distress: Feasibility of testing a Reiki intervention. J. Holist. Nurs..

[31] Tsang K.L., Carlson L.E., Olson K. (2007). Pilot Crossover Trial of Reiki Versus Rest for Treating Cancer-Related Fatigue. Integr. Cancer Ther..

[32] Richeson N.E., Spross J.A., Lutz K., Peng C. (2010). Effects of Reiki on Anxiety, Depression, Pain, and Physiological Factors in Community-Dwelling Older Adults. Res. Gerontol. Nurs..

[33] Shore A.G. (2004). Long-term effects of energetic healing on symptoms of psychological depression and self-perceived stress. Altern. Ther. Health Med..

[34] Erdogan Z., Cinar S. (2016). The Effect of Reiki on Depression in Elderly People Living in Nursing Home.

[35] Shiflett S.C., Nayak S., Bid C., Miles P., Agostinelli S. (2002). Effect of Reiki Treatments on Functional Recovery in Patients in Poststroke Rehabilitation: A Pilot Study. J. Altern. Complement. Med..

[36] Beard C., Stason W.B., Wang Q., Manola J., Dean-Clower E., Dusek J., DeCristofaro S., Webster A., Doherty-Gilman A.M., Rosenthal D.S. (2010). Effects of complementary therapies on clinical outcomes in patients being treated with radiation therapy for prostate cancer. Cancer.

[37] Joyce J., Herbison G.P. (2015). Reiki for depression and anxiety. Cochrane Database Syst. Rev..

[38] Berger L., Tavares M., Berger B. (2013). A Canadian Experience of Integrating Complementary Therapy in a Hospital Palliative Care Unit. J. Palliat. Med..

[39] Brill C., Kashurba M. (2001). Each Moment of Touch. Nurs. Adm. Q..

[40] Gill L. More Hospitals Offer Alternative Therapies for Mind, Body, Spirit. USA Today 2015. https://usatoday30.usatoday.com/news/health/2008–09–14-alternative-therapies_N.htm.

[41] Billot M., Jaglin P., Rainville P., Rigoard P., Langlois P., Cardinaud N., Tchalla A., Wood C. (2020). Hypnosis Program Effectiveness in a 12-week Home Care Intervention To Manage Chronic Pain in Elderly Women: A Pilot Trial. Clin. Ther..

[42] Dumain M., Jaglin P., Wood C., Rainville P., Pageaux B., Perrochon A., Lavallière M., Vendeuvre T., Romain D., Langlois P. (2021). Long-Term Efficacy of a Home-Care Hypnosis Program in Elderly Persons Suffering From Chronic Pain: A 12-Month Follow-Up. Pain Manag. Nurs..

[43] Chen Y.-J., Petrinec A., Stephenson P.S., Radziewicz R.M., Sheehan D. (2021). Home-Based Reiki by Informal Caregivers: A Mixed-Methods Pilot Study. Holist. Nurs. Pr..

